# Effect of exposure to COVID‐19 infodemic on infection‐preventive intentions among Korean adults

**DOI:** 10.1002/nop2.965

**Published:** 2021-06-19

**Authors:** Jeong‐Won Han, Junhee Park, Hanna Lee

**Affiliations:** ^1^ College of Nursing Science Kyung Hee University Seoul Korea; ^2^ Department of Nursing Dongnam Health University Gyeonggi‐do Korea; ^3^ Department of Nursing Gangneung‐Wonju National University Gangwon‐do Korea

**Keywords:** COVID‐19, infections, intention, knowledge, Republic of Korea

## Abstract

**Aim:**

This study determined the effect of exposure to coronavirus disease 2019 (COVID‐19) infodemic on infection‐preventive intentions among Korean adults.

**Design:**

This was a cross‐sectional study that used structural equation model.

**Methods:**

Data were collected between 14 April–7 July 2020 from 300 adults in their 20s to 60s residing in South Korea. Analysis was performed using SPSS 20.0 and AMOS 20.0.

**Results:**

Exposure to COVID‐19 infodemic had a direct effect on the reduction of COVID‐19‐related knowledge and personal preventive health intentions. COVID‐19‐related knowledge had a direct impact on increased perceived severity, perceived vulnerability, perceived self‐efficacy, and personal preventive health intentions. Perceived severity, perceived vulnerability, and perceived self‐efficacy had a direct effect on increasing preventive intentions for personal health; and perceived severity had a direct effect on preventive intentions for public health.

## 1 INTRODUCTION

The coronavirus 2019 (COVID‐19) pandemic is an ongoing global disease caused by the severe acute respiratory syndrome coronavirus 2 (SARS‐CoV‐2) (Wordometer, 2021). The new COVID‐19 cases increased for the ninth consecutive week, with nearly 5.7 million new cases reported in the last week—surpassing previous peaks (WHO, 2021a). The number of new deaths increased for the sixth consecutive week, with over 87,000 new deaths reported (WHO, 2021a). Overall, and until April 2021, 152,632,166 cases and 3,201,593 deaths had been confirmed across 222 countries, making it one of the deadliest pandemics in history (Wordometer, 2021). During an epidemic, early diagnosis and treatment of infected cases along with the protection of healthy individuals are of high concern (Wang & Zhang, 2020). However, in the 21st century, characterized by new lifestyles and fast transportation that contribute to the global spread of diseases, countries tend to face additional challenges in controlling epidemics. An “infodemic,” defined as a rapid spread of all kinds of information concerning a problem such that the solution is made more difficult (WHO, 2018), is also one such additional challenge, as the popular use of social media and communication technologies is rising (WHO, 2021b), and has the potential of accelerating the epidemic process by influencing and fragmenting social response (Kim et al., 2019). COVID‐19 appears to be a true social‐media infodemic compared to previous viral outbreaks. Indeed, there has been a spread of disinformation at an exceptional speed, creating an environment of amplified uncertainty that has fuelled anxiety and racism online and in person (Vaezi & Javanmard, 2020). At a time when COVID‐19 cases are continuing to rise, understanding how exposure to the COVID‐19 infodemic affects preventive intentions for personal and public health and controls psychological protection motivation factors is critical for forming a strategic response against the spread of the infection.

## 2 BACKGROUND

Problems caused by COVID‐19 have identified as a social issue; hence, both the current status of the pandemic and the methods used to prevent its spread are being widely circulated through the media. Information about COVID‐19 is being posted on the main pages of Internet portals and also being provided by the related organizations at national and local levels (KCDCP, 2020). Furthermore, information such as news reports on COVID‐19, prevention methods and health measures taken by the afflicted patients is being shared among users on social media (KCDCP, 2020). In particular, people are anxious due to the impossibility of curing the disease and the inability to receive accurate information related to the infection (Park et al., 2016). Thus, they look to reduce uncertainty by obtaining the maximum amount of infection‐related information from social media and various communication technologies (Park et al., 2016).

During a pandemic, people naturally tend to search for accurate, trustworthy information; however, due to the current infodemic, this information may be obscured as it is intermingled with misinformation (Greenspan & Loftus, 2020). Even before the COVID‐19 pandemic, researching on the Internet was the most common way to obtain health information (Tan & Goonawardena, 2017). However, since the quality and content of health information provided on the Internet can vary, the more individuals collect Internet‐based COVID‐19 information, the greater the negative impact on their psychological well‐being (Pramukti et al., 2020). Due to cognitive limitations, it is difficult for people to correctly perceive, remember, reason, judge, decide and act on information as complex and uncertain as the one pertaining COVID‐19, resulting in the making of irrational decisions (Tversky & Kahneman, 1974). Sharing and accumulating of such inaccurate information may promote the spread of the infection by changing the perception and behaviour of people about the infection (Song & Kim, 2017).

Infection‐related preventive behaviour can be explained through the protection motivation theory of Rogers (1975), which states that when people are exposed to disease‐related information, they judge whether the disease is fearful or dangerous to them based on such information. When a disease is perceived as a threat, it triggers a protection motivation in the individual, which in turn induces behavioural change to prevent the disease. In particular, the protection motivation theory emphasizes that individual behavioural changes are mediated and controlled by information users' psychological factors (perceived threats, severity, self‐efficacy, among others), rather than occurring as simple behavioural changes in response to threat messages. In previous studies, fear of COVID‐19 was an important mediating factor of perceived health status, insomnia, mental health and COVID‐19 preventive behaviour (Ahorsu et al., 2020). In addition, a study using Iranian participants reported that psychological factors, such as coping planning and action planning, have the most influence on the motivation and will to take action to prevent COVID‐19 (Lin, Imani, et al., 2020). Exposure to disease information through social media and communication technologies affects severity, which refers to the degree of harm caused by the threat of disease; vulnerability, which refers to the opportunity that one could be exposed to the infection; and response efficacy and self‐efficacy, which appraise the effectiveness of responding to the threat (Kim, 2010). Most people in modern society are exposed to information on health and disease through social media, through which they accumulate knowledge on responding to health and diseases (Case et al., 2004). Therefore, increased exposure to incorrect information creates false knowledge, which can lead to the collapse of disease‐related preventive intentions through distorted cognitive processing of the disease (Case et al., 2004). Thus, increasing an individual's confidence in the source of COVID‐19 information can be a way to increase preventive behaviour (Chang et al., 2020). Therefore, this study aimed to examine the effect of exposure to COVID‐19 infodemic on infection‐preventive intentions among Korean adults.

## 3 METHOD

### 3.1 Design

This study conducted a path analysis to determine the effect of exposure to COVID‐19 infodemic on infection‐preventive intentions among Korean adults.

### 3.2 Participants

The participants were recruited through convenience sampling; the inclusion criteria were as follows: (a) adults in their 20s to 60s; (b) residing in Busan, Daegu, Gyeongsang‐do (Daegu, Ulsan, Jinju, Masan and Geojedo), and Gyeonggi‐do, where the number of confirmed COVID‐19 cases was high at the time of the study; (c) not engaged in health care (Hospital, Clinic, Health Center, etc.); and (d) work in a setting not directly exposed to COVID‐19. The use of the maximum‐likelihood estimation method in a structural equation model suggested a sample size of at least 150, with a sample size between 200–400 being preferable (Yu, 2012). Accordingly, a sample size of 300 was determined to be suitable for conducting this study.

### 3.3 Measurements

#### 3.3.1 Exposure to COVID‐19 Infodemic

The tool for assessing exposure to COVID‐19 infodemic was developed by the authors of this study based on the contents of “COVID‐19 Information” (KCDCP, 2020) released by the Ministry of Health and Welfare and the Centers for Disease Control and Prevention. A website was developed to present the grounds for incorrect information and provide accurate information to the public through its “COVID‐19 Fact and Issue Check” section. After developing the tool items, an expert group—consisting of three nursing managers and nursing professors working in the infection control room at K Hospital in S City—was selected to verify the content validity of the items from 30 March–2 April 2020. Each question of the tool was evaluated on a 4‐point Likert scale consisting of the options: “very appropriate” (4 points), “appropriate” (3 points), “not appropriate” (2 points) and “not at all appropriate” (1 point). The content validity index was calculated to be 1.0 for all the items. In addition, the items were checked for readability and understandability by five adults in their 20s, 30s, 40s, 50s and 60s. The tool comprised 21 items, mainly related to incorrect knowledge about COVID‐19 prevention. For instance, items enquired whether foods such as kimchi, garlic, water, mineral supplements, ginger, balloon flower, alcohol, curry, sesame oil, sugar and spinach prevent COVID‐19; and whether applying antiphlamine ointment and using salt water prevents coronavirus. In addition, there were items on whether boiling clothes in bleach, microwaving money, attaching silver or taking antibiotics help against coronavirus. Some items assessed information about incorrect infection routes and diagnosis of COVID‐19. Each item asked the question “Have you heard or seen this COVID‐19‐related information on the Internet, TV, radio, SNS, etc.?” to which respondents were instructed to choose either “Yes” or “No.” With a score of 1 for “Yes” and 0 for “No,” scores ranged from 0–21, with a higher score indicating greater exposure to the COVID‐19 infodemic. The reliability of the tool was found to be 0.850 under KR‐20 (Kuder‐Richardson formula 20).

#### 3.3.2 Knowledge of COVID‐19

The tool for measuring the knowledge of COVID‐19 was developed by the authors of this study based on the description of “COVID‐19 Information” (KCDCP, 2020) released by the Ministry of Health and Welfare and the Centers for Disease Control and Prevention. After developing the tool items, an expert group consisting of three nursing managers and nursing professors working in the infection control room at K Hospital in S City was selected to verify the content validity of the items from 30 March–2 April 2020. Each question was evaluated on a 4‐point Likert scale consisting of the options: “very appropriate” (4 points), “appropriate” (3 points), “not appropriate” (2 points) and “not at all appropriate” (1 point). The content validity index was calculated to be 1.0 for all items. In addition, the items were checked for readability and understandability by three adult males in their 30s and 40s. The questionnaire consisted of 11 items, which assessed knowledge about transmission route, incubation period, symptoms, fatality rate and prevention method; and respondents were asked to choose among “Correct,” “Incorrect” and “Don't know.” With a score of 1 for “Correct” and 0 for “Incorrect” and “Don't know,” scores ranged from 0–11, and a higher score indicated greater knowledge. The reliability of the tool was found to be 0.784 under KR‐20.

#### 3.3.3 Psychological mechanisms

A modified version of Kim's (2010) measurement tool was used to assess psychological mechanisms, which consisted of four factors. Perceived severity included three questions on the harm that COVID‐19 will inflict on individuals, society and the world. Perceived vulnerability included three questions on the opportunity that one could contract COVID‐19 at restaurants, in public transportation and in the community. Response efficacy included six questions concerning the “national prevention rules” including hand washing, covering mouth with sleeve when coughing, not touching eyes, nose and mouth with unwashed hands, avoiding contact with symptomatic patients, wearing masks and refraining from visiting crowded places, as stated in the “COVID‐19 Preventive Behavioral Rules” provided by the Korea Centers for Disease Control and Prevention. Self‐efficacy assessed the belief that one will be able to overcome COVID‐19 through preventive measures. All four factors were measured on a 5‐point scale ranging from 1 indicating “not at all”–5 indicating “strongly agree.” In Kim’s (2010) study, the reliability of the tools, as indicated through Cronbach's alpha values, was 0.76, 0.84, 0.76 and 0.75 for perceived severity, perceived vulnerability, response efficacy and self‐efficacy, respectively. In this study, the Cronbach's alpha was 0.81, 0.85, 0.82 and 0.91 for perceived severity, perceived vulnerability, response efficacy and self‐efficacy, respectively.

#### 3.3.4 Preventive intentions

In this study, Kim’s (2010) tool on infection‐preventive behavioural intentions was used to measure preventive intentions, which included the sub‐areas of preventive intentions for personal and public health. Preventive intentions for personal health protection were measured on a 5‐point scale ranging from 1 indicating “not at all”–5 indicating “strongly agree” for three items concerning personal hygiene, including wearing masks, not going to crowded places, and working or studying from home. Preventive intentions for public health protection were measured on a 5‐point scale ranging from 1 indicating “not at all”–5 indicating “strongly agree” for three items including refraining from going outside, wearing masks, and using own vehicles to visit medical institutions. The reliability of the tool in Kim’s (2010) study, as indicated through Cronbach's alpha values, was 0.70 for preventive intentions for personal health protection and 0.76 for preventive intentions for public health protection, whereas they were 0.77 and 0.76, respectively, in this study.

### 3.4 Data collection and procedure

This study was conducted after receiving approval from the institutional review board of the researchers’ affiliated institution. Considering the COVID‐19 situation, the questionnaire was not administered to confirmed or suspected COVID‐19 patients or those in quarantine. The questionnaire was sent by mail, and the participants were asked to respond to the questionnaire after completing the enclosed participation consent form. It was stated in the explanation that the survey results would not be used for purposes other than the designated research purpose, that participation may be withdrawn at any time during the research process should the participants want to opt out, and that participation would be confidential and processed anonymously. All data were left anonymous to maintain confidentiality.

### 3.5 Data analysis

Data collected for this study were analysed with SPSS 21.0 (SPSS Korea Data Solution Inc.) and AMOS 21.0 (SPSS Korea Data Solution Inc.). General characteristics of the participants and research variables were analysed using descriptive statistics. For the measurement tools, confirmatory factor analysis (CFA) was conducted; construct reliability and average variance extracted (AVE) were used to examine convergent validity, which tests the degree to which various measures for a single construct are related. Correlation coefficients and AVE values were used to test discriminant validity, which shows the degree to which measures for different constructs are unrelated. The reliability of the measurement tools was confirmed with KR‐20 and Cronbach's alpha. Normality of the sample was verified using mean, standard deviation, skewness and kurtosis, and the correlation between measured variables was verified with Pearson's correlation coefficients. To test the goodness‐of‐fit, chi‐square (χ^2^), chi‐square/*df* (χ2/*df* ≤ 3.00), adjusted goodness‐of‐fit index (AGFI ≥ 0.90), goodness‐of‐fit index (GFI ≥ 0.90), comparative fit index (CFI ≥ 0.90), standardized root mean square residual (SRMR ≤ 0.05), root mean square error of approximation (RMSEA ≤ 0.10), normed fit index (NFI ≥ 0.90) and Tucker‐Lewis Index (TLI ≥ 0.90) were used. The model goodness‐of‐fit reference value is a suitable model when the values of GFI, AGFI, CFI and TLI are more than 0.90 and SRMR is < 0.05 (Hu & Bentler, 1998) and when the value of RMSEA is < 0.10 (MacCallum et al., 1996). Bootstrapping was used to verify the significance of the model's indirect and total effects. In this study, the significance of the indirect effect was confirmed by applying the 1,000 bootstrapping repetitions and Bias corrected (BC) method.

### 3.6 Ethical considerations

This study was conducted after receiving approval from the institutional review board of the researchers’ affiliated institution (****IRB‐****‐*). The survey was performed after obtaining consent.

## 4 RESULTS

### 4.1 General characteristics of the participants

The research participants consisted of 148 (49.3%) male and 152 (50.7%) female participants. The mean age of the participants was 41.85 ± 14.11, and, as for their academic background, 81 (27.0%) were high school graduates, 34 (11.3%) had an associate degree, and 185 (61.7%) had a bachelor's degree or higher. Twelve (4.0%) participants had undergone COVID‐19 screening tests, and 288 (96.0%) had not. The most frequent source of COVID‐19‐related information was the Internet for 139 participants (46.3%), followed by television for 118 participants (39.3%) (Table 1).

**TABLE 1:** General characteristics

Variables	Categories	*N* (%)	M ± SD
Gender	Male	148 (49.3)	
	Female	152 (50.7)	
Age (year)	<30	62 (20.7)	41.85 ± 14.11
	30–39	76 (25.3)	
	40–49	64 (21.3)	
	50–59	47 (15.7)	
	60–69	51 (17.0)	
Level of education	Under high school	81 (27.0)	
	College	34 (11.3)	
	Graduate school	185 (61.7)	
Religion	Christianity	36 (12.0)	
	Buddhism	49 (16.3)	
	Catholicism	99 (33.0)	
	None	116 (38.7)	
Marital status	Married	213 (71.0)	
	Not married	84 (28.0)	
	Other	3 (1.0)	
Number of children	0	46 (15.3)	
	1	75 (25.0)	
	2	155 (51.7)	
	≥3	24 (8.0)	
Residence	Gyeonggi‐do	195 (65.0)	
	Daegu	33 (11.0)	
	Busan	52 (17.3)	
	Gyeongsang‐do	20 (6.7)	
Job	Yes	210 (70.0)	
	None	90 (30.0)	
COVID−19 screening test	Yes	12 (4.0)	
	None	288 (96.0)	
Confirmed cases of COVID−19	Family or cohab	2 (0.7)	
	Neighbour	18 (6.0)	
	Colleague at work	4 (1.3)	
	None	276 (92.0)	
Route on information related to COVID−19	Family	1 (0.3)	
	Friend	5 (1.7)	
	TV	118 (39.3)	
	Radio	6 (2.0)	
	Internet	139 (46.3)	
	SNS	29 (9.7)	
	Other	2 (0.7)	

Abbreviations: M, Mean; *SD*, Standard deviation; SNS, Social network services.

### 4.2 Descriptive statistics, convergent validity and discriminant validity for measured variables

Table 2 shows the descriptive values of the measurement variables. All measured variables for this study satisfied normality in the univariate normality test. However, multivariate normality was not satisfied at a significance level of 0.05, as a multivariate normality test yielded a multivariate kurtosis value that exceeded the threshold of 18.72 at 26.90. Therefore, bootstrapping was used for structural equation modelling and significance testing. CFA was conducted in this study to test convergent and discriminant validity. Factor loadings for all items were found to exceed the threshold of 0.6, and the CR (t) threshold of 1.96 when analysing convergent validity. In addition, conditions for convergent validity were satisfied with the average variance extracted (AVE) ranging from 0.75–0.95, which is above the threshold of 0.50, and construct reliability ranging from 0.85–0.99, which is above the threshold of 0.70. Discriminant validity was established as the AVE value of latent variables was found to be larger than the squared value of the correlation coefficient among them.

**TABLE 2:** Correlations among the variables

Variables		M ± SD	X1	X2	X3	X4	X5	X6	X7	X8
X1	Exposure level of infodemics	2.36 ± 3.08	1							
X2	Knowledge related to COVID−19	10.20 ± 1.12	0.20*	1						
X3	Perceived severity	4.67 ± 0.49	0.39*	0.23*	1					
X4	Perceived vulnerability	4.39 ± 0.62	0.32*	0.29*	0.54*	1				
X5	Perceived coping efficacy	4.20 ± 0.61	0.30*	0.35*	0.47*	0.51*	1			
X6	Perceived self‐efficacy	3.89 ± 0.78	0.35*	0.30*	0.32*	0.35*	0.71*	1		
X7	Individual health prevention intention	4.23 ± 0.65	0.40*	0.42*	0.28*	0.31*	0.30*	0.44*	1	
X8	Public health prevention intention	4.68 ± 0.54	0.38*	0.37*	0.43*	0.31*	0.31*	0.23*	0.43*	1
KR 20/ Cronbach’ α		0.85	0.78	0.81	0.85	0.82	0.91	0.77	0.76	

Abbreviations: M, Mean; *SD*, Standard deviation; KR 20, Kuder‐Richardson formula 20.

* *p* < .05.

### 4.3 Effect of COVID‐19 infodemic on preventive intentions

**FIGURE 1:**
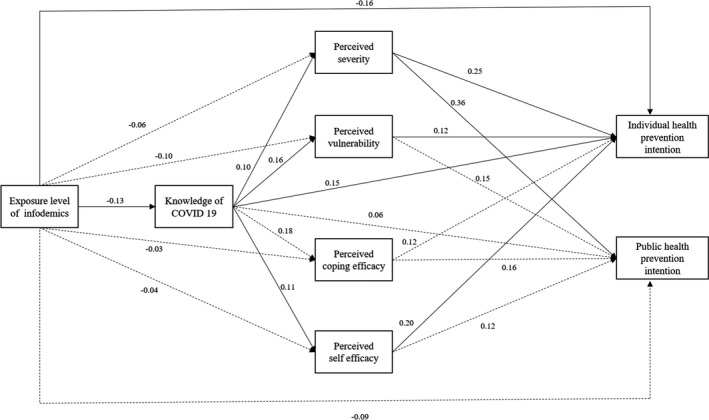
The path diagram of the model

Evaluating the goodness‐of‐fit for the research model resulted in χ^2^ = 19.80, *df* = 8, χ^2^/*df* = 2.48, GFI = 0.99, AGFI = 0.94, NFI = 0.97, CFI = 0.98, TLI = 0.97, SRMR = 0.05 and RMSEA = 0.06. Exposure to COVID‐19 infodemic was found to have a direct effect on COVID‐19‐related knowledge (β = −0.13, *p* = .016) and personal preventive health intentions (β = −0.16, *p* = .002). COVID‐19‐related knowledge was found to have a direct effect on perceived severity (β = 0.10, *p* = .025), vulnerability (β = 0.16, *p* = .040), self‐efficacy (β = 0.11, *p *= .041) and personal preventive health intentions (β = 0.15, *p *= .006). Perceived severity (β = 0.25, *p *< .001), vulnerability (β = 0.12, *p *= .026) and self‐efficacy (β = 0.20, *p *= .005) were found to have a direct effect on personal preventive health intentions, and perceived severity (β = 0.36, *p *< .001) was found to have a direct effect on public health preventive intentions. Furthermore, exposure to the infodemic was found to have an indirect effect on perceived severity, vulnerability, self‐efficacy and preventive intentions. COVID‐19‐related knowledge was shown to have an indirect effect on preventive intentions (Table 3) (Figure 1).

**TABLE 3:** Effects of predictive variables in the model

Exogenous variables	Endogenous variables	β	CR	*p*	Direct effects (*p*)	Indirect effects (*p*)	Total effects (*p*)
E	K	−0.13	−2.42	0.016	−0.13 (0.016)	–	−0.13 (0.016)
K	PS	0.10	2.18	0.025	0.10 (0.025)	–	0.10 (0.025)
E	PS	−0.06	−1.087	0.276	−0.06 (0.276)	−0.10 (0.045)	−0.16 (0.036)
K	PV	0.16	2.07	0.040	0.16 (0.040)	–	0.16 (0.040)
E	PV	−0.10	−1.711	0.087	−0.10 (0.087)	−0.19 (0.013)	−0.29 (0.009)
K	PCE	0.18	1.49	0.134	0.18 (0.134)	–	0.18 (0.134)
E	PCE	−0.03	−0.514	0.607	−0.03 (0.607)	−0.11 (0.113)	−0.14 (0.727)
K	PSE	0.11	2.05	0.041	0.11 (0.041)	–	0.11 (0.041)
E	PSE	−0.04	−0.649	0.516	−0.04 (0.516)	−0.14 (0.013)	−0.18 (0.097)
PS	IHPI	0.25	4.21	<0.001	0.25 (<0.001)	–	0.25 (<0.001)
PV	IHPI	0.12	2.04	0.026	0.12 (0.026)	–	0.12 (0.026)
PCE	IHPI	0.12	0.26	0.791	0.12 (0.791)	–	0.12 (0.791)
PSE	IHPI	0.20	2.78	0.005	0.20 (0.005)	–	0.20 (0.005)
E	IHPI	−0.16	−3.09	0.002	−0.16 (0.002)	−0.13 (0.006)	−0.28 (0.018)
K	IHPI	0.15	2.77	0.006	0.15 (0.006)	0.25 (0.004)	0.40 (0.040)
PS	PHPI	0.36	6.01	<0.001	0.36 (<0.001)	–	0.36 (<0.001)
PV	PHPI	0.15	0.75	0.452	0.15 (0.452)	–	0.15 (0.452)
PCE	PHPI	0.16	0.80	0.423	0.16 (0.423)	–	0.16 (0.423)
PSE	PHPI	0.12	0.35	0.724	0.12 (0.724)	–	0.12 (0.724)
E	PHPI	−0.09	−1.63	0.103	−0.09 (0.103)	−0.10 (0.030)	−0.19 (0.034)
K	PHPI	0.06	1.09	0.274	0.06 (0.274)	0.14 (0.024)	0.20 (0.108)

Abbreviations: E, Exposure of infodemics; K, Knowledge related to COVID‐19; PS, Perceived severity; PV, Perceived vulnerability; PCE, Perceived coping efficacy; PSE, Perceived self‐efficacy; IHPI, Individual health prevention intention; PHPI, Public health prevention intention.

## 5 DISCUSSION

This study's results present important implications. First, participants’ exposure to the COVID‐19 infodemic was found to decrease both knowledge about COVID‐19 and personal preventive health intentions. This is similar to research findings (Hua & Shaw, 2020; Karimi & Gamrell, 2020) that claim increased exposure to infodemic may distort an individual's knowledge of COVID‐19. This also supports the results of several previous studies (Abd‐Alrazaq et al., 2020; Liew et al., 2020; Pulido et al., 2020a; Pulido, Villarejo, et al., 2020) that show that exposure to infodemic has a statistically significant impact on public health communications and reduces infection‐preventive intentions. Misinformation about COVID‐19 can not only reduce infection‐preventive intentions, but can also lead to ineffective responses that spread the infection further and cause social anxiety (Pennycook et al., 2020); hence, it is important for people to share and obtain correct information in situations involving infectious diseases. In particular, due to the recent increase in dependence on social media and communication technologies as a major means of seeking information, most information is being spread online (Guille et al., 2013). This suggests an urgent need for social interventions to improve the quality of information available online. Many recent studies (Frenkel et al., 2020; Russonello, 2020) have found that infodemics are being spread via the Internet, including social media. Also, since problematic social media use is significantly correlated with psychological distress, to minimize the spread of COVID‐19 infection, healthcare providers have appropriate online campaigns to eliminate people's fear of COVID‐19 and reduce misunderstandings about it (Lin et al., 2020).

Furthermore, the results of this study also indicate that the Internet and social network services make up more than half of all sources of COVID‐19‐related information. Individuals need to take responsibility for online information, and there needs to be comprehensive ethical education on the harms and risks that may be caused by an infodemic. It is also necessary to consider institutional mechanisms that impose responsibility for the spread of fake news. In Korea, efforts are being made to establish accuracy on COVID‐19‐related information through a Q & A board on the homepage of the Ministry of Health and Welfare (KCDCP, 2020). However, in addition to media based on unilateral communication, the government should establish and promote a system for convenient and quick delivery of correct information through means such as social media that allows for bilateral communication. Moreover, it is necessary to consider a delivery method that can be understood at the level of public literacy, since correct information should not only be used, but also be comprehended and accepted.

Second, COVID‐19‐related knowledge was found to have a direct impact on perceived severity, perceived vulnerability, and perceived self‐efficacy. This reaffirms the results of previous studies in which knowledge was found to affect behavioural intentions through similar psychological mechanisms (Ahorsu et al., 2020; Lin, Imani, et al., 2020; Rippetoe & Rogers, 1987; Tanner et al., 1991). This is consistent with a previous study (Woo, 2007) in which the perception of fear and response knowledge about diseases were found to increase protection motivation against diseases, and consequently increase behavioural intentions to promote health. Before taking preventive action against a given disease, people weigh the benefits and disadvantages of such action (Rosenstock et al., 1998). In this process, greater knowledge of the disease can lead to a more serious consideration of the severity, vulnerability and self‐efficacy related to it (Kim et al., 2010). Thus, knowledge of COVID‐19 can be regarded as affecting the participants’ perceived severity, perceived vulnerability and perceived self‐efficacy by becoming the basis on which the participants judge the benefits and disadvantages of their actions before engaging in preventive behaviour. Additionally, exposure to the COVID‐19 infodemic was found to have an indirect effect on participants’ perceived severity, perceived vulnerability and perceived self‐efficacy. This indirect effect of the infodemic exposure can be attributed to the fact that individuals seek and accumulate knowledge when looking for information about a disease. However, the contents of the infodemic based in South Korea also include folk remedies for various diseases that can be found in Korean culture. Therefore, greater exposure to such infodemic tends to result in diseases being perceived as light and easy to cope with because of the familiarity of information, thereby lowering the extent of an individual's perceived severity, vulnerability and self‐efficacy. As such, in order to increase the preventive intentions against infectious diseases such as COVID‐19, it is critical to create a method for accumulating correct knowledge of such diseases. At the same time, it is also necessary to increase the psychological mechanisms of individuals about infectious diseases by minimizing their exposure to infodemics or establishing a verification system for distorted information that can filter out infodemics.

Third, the results indicated that the perceived severity of a participant had a direct effect on the preventive intentions for public health. Moreover, perceived severity, perceived vulnerability and perceived self‐efficacy were found to have an influence on the preventive intentions for personal health. In particular, this study showed that perceived severity had a greater effect on preventive intentions for public health than on preventive intentions for personal health. This is similar to the results of a previous study (Jordan et al., 2020), in which the emphasis on COVID‐19 as a pandemic and the prolonged duration of the disease made promotional material focusing on public health, such as “do not spread the coronavirus” more effective than that focusing on personal health, such as “do not contract the coronavirus.” These results suggest that in the case of infectious diseases, providing accurate information, which increases the severity of the disease as perceived by the people, could lead to heightened infection‐preventive intentions. It is also worth noting that the infodemic had a direct effect on lowering preventive intentions for personal health in this study. As explained above, this could be attributed to the fact that infodemics originating in South Korea include aspects of folk remedies, and extended exposure to such infodemics could result in a lighter and less severe perception of a disease than it actually is. In particular, people must be made to understand and regulate the characteristics of uncertainty as during the outbreak of a new infectious disease such as COVID‐19, it is difficult even for health professionals to accurately predict the disease's course due to a lack of experience in dealing with the disease. Furthermore, in addition to establishing a system for preventing and responding to infodemics at the governmental or institutional level, individuals exposed to infodemics must also take the time to verify online information rather than accepting it immediately without question.

## 6 CONCLUSION

This study was conducted to determine the effect of exposure to the COVID‐19 infodemic on infection‐preventive intentions among Korean adults. The results indicate that controlling exposure to the COVID‐19 infodemic affects the knowledge and psychological mechanisms related to infectious diseases among adults, indicating that it could be an effective method to increase infection‐preventive intentions among adults. However, the study also has some limitations. First, the investigation in this study is self‐reported, so there is an opportunity that a single rater bias may have occurred. Second, because this study used a cross‐sectional design, causal inference may be weak. Third, people who might have been infected with SARS‐COV‐2 or have been in close contact with positive cases are expected to have a higher and more accurate understanding of COVID‐19; however, this study has not considered this opportunity. Finally, since this study was conducted before the end of the COVID‐19 pandemic, it could not obtain a larger sample due to the risk of infection, so the sample was not representative. Also, exposure to COVID‐19 could differ across age groups, regions and exposure routes; hence, we suggest that this aspect be considered when conducting future studies.

## CONFLICT OF INTEREST

The authors declare that they have no conflict of interest.

## AUTHOR CONTRIBUTIONS

Han Jeong‐Won: Conceptualization, methodology, software, validation, formal analysis, writing ‐ original draft preparation, writing ‐ review and editing. Park Junhee: Data curation, investigation, writing ‐ original draft preparation, writing ‐ review and editing. Lee Hanna: Visualization, investigation, writing ‐ original draft preparation, writing ‐ review and editing.

## ETHICAL CONSIDERATION AND CONSENT TO PARTICIPATE

This study was conducted after receiving approval from the institutional review board of the researchers’ affiliated institution (GWNUIRB‐2020–7). The survey was performed after the obtaining consent.

## Data Availability

No data are available in online. All supporting data can be provided upon request to the authors.
